# IgM- and IgA-Enriched Polyclonal Intravenous Immunoglobulin Therapy in Sepsis: A Single-Center Retrospective Cohort Study

**DOI:** 10.3390/jcm15197731

**Published:** 2026-10-08

**Authors:** Hakan Yılmaz, Melek Tulunay, Ali Abbas Yılmaz

**Affiliations:** Department of Anesthesiology and Reanimation, Faculty of Medicine, Ankara University, 06230 Ankara, Türkiyedoktorabbas@yahoo.com (A.A.Y.)

**Keywords:** sepsis, septic shock, intravenous immunoglobulin, IgM- and IgA-enriched immunoglobulin, IgGAM, initial empiric antibiotic therapy, organ dysfunction, mortality

## Abstract

**Background/Objectives**: Evidence for adjunctive intravenous immunoglobulin therapy in sepsis remains conflicting. This study evaluated the association of IgM- and IgA-enriched polyclonal intravenous immunoglobulin (IgGAM) therapy with clinical outcomes, including post hoc analyses according to initial empiric antibiotic therapy (IEAT) appropriateness. **Methods**: This single-center retrospective cohort study included 50 adults with sepsis or septic shock according to Sepsis-3 criteria, treated between November 2006 and March 2010. Twenty-five patients received adjunctive IgGAM plus standard sepsis management, and 25 received standard management alone. Primary outcomes were 28-day intensive care unit (ICU) mortality, overall ICU mortality, and in-hospital mortality. **Results**: Overall, 28-day ICU mortality was 28.0% with IgGAM and 32.0% with control (relative risk, 0.88; 95% confidence interval [CI], 0.37–2.05; *p* = 0.758); other mortality outcomes did not differ significantly. Kaplan–Meier survival distributions also did not differ significantly (log-rank *p* = 0.208). During the ICU stay, vasopressor therapy was required in 68% of the IgGAM group and 92% of the control group (*p* = 0.034); corresponding study day 1 requirements were 60% and 76% (*p* = 0.225). In the complete-case longitudinal analysis (IgGAM group, *n* = 20; control group, *n* = 25), SOFA scores decreased significantly over time within the IgGAM group (*p* = 0.017), whereas no significant within-group change was observed in the control group (*p* = 0.616). In the post hoc appropriate-IEAT subgroup, 28-day ICU mortality was 0.0% versus 36.8% (*p* = 0.026), in-hospital mortality was 25.0% versus 63.2% (*p* = 0.038), and Kaplan–Meier survival distributions differed significantly (log-rank *p* = 0.004). The 60-day restricted mean survival time was 58.1 versus 38.4 days, with a difference of 19.8 days (95% CI, 11.1–28.4). **Conclusions**: Adjunctive IgGAM therapy was not associated with significant differences in mortality overall. Findings in the post hoc appropriate-IEAT subgroup were hypothesis-generating and should be interpreted cautiously given potential selection bias, residual confounding, and multiple comparisons. Prospective evaluation within optimized sepsis care is warranted.

## 1. Introduction

Sepsis is a life-threatening clinical syndrome characterized by organ dysfunction caused by a dysregulated host response to infection. In 2017, an estimated 48.9 million incident cases of sepsis and 11.0 million sepsis-related deaths occurred worldwide, representing 19.7% of all global deaths [1]. A systematic review and meta-analysis of studies from Europe and North America estimated that septic shock was present at ICU admission in 10.4% of patients, with a pooled ICU mortality of 37.3% [2].

Sepsis is characterized by concurrent hyperinflammatory and immunosuppressive responses that may result in sustained immune dysfunction [3]. In a prospective multicenter study, serum IgM levels decreased with progression from severe sepsis to septic shock and were lower in non-survivors [4]. In addition, the combined presence of low endogenous plasma levels of IgG1, IgM, and IgA has been associated with increased mortality in patients with severe sepsis or septic shock [5]. Intravenous immunoglobulin (IVIG) may modulate both proinflammatory and anti-inflammatory immune responses through multiple mechanisms, including neutralization of exo- and endotoxins, enhanced pathogen clearance through opsonization, modulation of complement and cytokine cascades, attenuation of Fc receptor-mediated phagocyte activation, and potential attenuation of lymphocyte apoptosis [6,7,8,9,10,11,12]. Pentaglobin^®^, an IgM- and IgA-enriched polyclonal intravenous immunoglobulin (IgGAM) preparation, has been shown to contain higher concentrations of lipopolysaccharide-specific antibodies in its IgM fraction than pure IgG preparations, supporting a potential role for IgM-class antibodies in immune responses to bacterial lipopolysaccharide [13]. Furthermore, IgM- and/or IgA-enriched immunoglobulin preparations have shown greater neutralizing activity against streptococcal pyrogenic exotoxin A than pure IgG preparations [14]. These findings have drawn attention to IVIG, particularly IgGAM preparations, as adjunctive immunomodulatory therapy in sepsis.

However, clinical evidence regarding the efficacy of IVIG in sepsis remains conflicting. One meta-analysis reported a survival benefit in patients with sepsis who received polyclonal IVIG compared with placebo or no intervention [15]. This benefit was not confirmed in another meta-analysis when analyses were restricted to high-quality studies [16]. In addition, a randomized controlled trial (RCT) showed that IVIG did not reduce mortality [17]. Both the 2021 and 2026 Surviving Sepsis Campaign guidelines suggest against the use of IVIG in adults with sepsis or septic shock [18,19]. Accordingly, recent work has emphasized patient stratification and personalized approaches to immunomodulatory therapy in sepsis, including efforts to identify subgroups in whom IVIG may warrant further evaluation [20,21,22,23,24,25].

The primary objective of this retrospective study was to evaluate the association of adjunctive IgGAM therapy with 28-day ICU mortality, overall ICU mortality, and in-hospital mortality in patients with sepsis or septic shock. Secondary objectives were to assess survival time, disease severity, organ dysfunction, and organ support requirements. Outcomes were further explored according to IEAT appropriateness in post hoc subgroup analyses.

## 2. Materials and Methods

### 2.1. Study Design, Data Source, and Ethical Approval

This single-center retrospective cohort study was conducted in the tertiary-care ICU of the Department of Anesthesiology and Reanimation at Ibni Sina Hospital, Ankara University Faculty of Medicine. Data were obtained from the hospital information management system and patient medical records.

The study was conducted in accordance with the principles of the Declaration of Helsinki. The study protocol was approved by the Human Research Ethics Committee of Ankara University Faculty of Medicine (Approval No. İ8-531-20; 18 September 2020). Because of the retrospective design and use of de-identified patient data, the requirement for individual informed consent was waived.

### 2.2. Study Population

The day of sepsis or septic shock diagnosis was defined as study day 1. Sepsis and septic shock were retrospectively defined according to Sepsis-3 criteria. Sepsis was defined as suspected or documented infection accompanied by an acute increase of ≥2 points in the Sequential Organ Failure Assessment (SOFA) score. Septic shock was diagnosed in patients who, despite adequate fluid resuscitation, required vasopressor therapy to maintain a mean arterial pressure (MAP) of ≥65 mmHg and had a serum lactate level > 2.0 mmol/L.

Adult patients admitted to the ICU with sepsis or septic shock between November 2006 and March 2010 were eligible for inclusion. Patients younger than 18 years and those with malignancy were excluded. A total of 50 patients met the inclusion criteria. Twenty-five patients had received adjunctive IgGAM for sepsis or septic shock beginning on study day 1, in addition to standard sepsis management, and constituted the IgGAM group. IgGAM was not administered for other indications in these patients. During the study period, IgGAM use was not protocolized, and no predefined eligibility criteria or treatment thresholds were applied. Treatment decisions were made individually by the senior intensivist supervising the ICU, based on clinical judgment informed by each patient’s clinical course and response to standard sepsis management. Pentaglobin^®^ (Biotest AG, Dreieich, Germany) was administered intravenously over 72 h at a rate of 0.4 mL/kg/h for the first 3.5 h and 0.2 mL/kg/h for the subsequent 68.5 h. The control group comprised 25 patients who received standard sepsis management alone from study day 1 onward and did not receive IgGAM during the ICU stay (Supplementary Figure S1).

### 2.3. Data Collection and Definitions

Age, sex, body weight, ICU admission type, admission source, pre-ICU location, and comorbidities were extracted from medical records. ICU admission type was classified as medical or surgical. Admission source was categorized as the same hospital or an external hospital, and pre-ICU location was categorized as the ward, another ICU, or the operating room. Recorded comorbidities included hypertension, coronary artery disease, congestive heart failure, chronic obstructive pulmonary disease, diabetes mellitus, chronic kidney disease, and chronic liver disease.

Data collected on study day 1 included sepsis stage, Acute Physiology and Chronic Health Evaluation II (APACHE II) score, SOFA score, vasopressor requirement, hemodynamic variables, laboratory parameters, and arterial blood gas (ABG) values. Sepsis stage was classified as sepsis or septic shock. APACHE II and SOFA scores were also calculated on study days 2, 3, 5, 7, 10, and 14. Hemodynamic variables included MAP and central venous pressure (CVP). Laboratory parameters included white blood cell count, platelet count, creatinine, total bilirubin, C-reactive protein, procalcitonin, international normalized ratio, and activated partial thromboplastin time. ABG variables included the PaO_2_/FiO_2_ ratio, lactate, bicarbonate, and base deficit.

During the study period, standard sepsis management included early fluid resuscitation, vasopressor therapy when required, timely initiation of broad-spectrum antibiotics followed by de-escalation based on culture results and antimicrobial susceptibility testing, and source control when indicated. Organ support therapies included lung-protective invasive mechanical ventilation (IMV) and renal replacement therapy (RRT) when required. RRT modalities included intermittent hemodialysis, continuous hemodialysis, and continuous hemofiltration. Other supportive therapies included intravenous hydrocortisone for refractory shock, low-molecular-weight heparin (LMWH) prophylaxis, and therapeutic plasma exchange (TPE) when indicated.

Cases were classified as culture-positive or culture-negative according to culture results obtained from blood, endotracheal aspirate, bronchoalveolar lavage, urine, catheter tip, wound, and drainage fluid samples. Identified microorganisms were classified as Gram-negative bacteria, Gram-positive bacteria, or fungi. Infection sites were categorized as pulmonary, intra-abdominal, urinary, catheter-related bacteremia, or primary bacteremia. Infections present at hospital admission or diagnosed within the first 48 h thereafter were classified as community-acquired, whereas infections diagnosed more than 48 h after admission or related to previous hospitalization were classified as hospital-acquired.

Initial empiric antibiotic therapy (IEAT) was defined as antibiotic therapy initiated within 3 h of sepsis or septic shock diagnosis. In culture-positive cases, IEAT was considered appropriate if the empiric regimen provided in vitro activity against all later-identified pathogens. For intra-abdominal infections, adequate anaerobic coverage was also required. IEAT was classified as inappropriate if these criteria were not met.

### 2.4. Outcomes

The primary outcomes were 28-day ICU mortality, overall ICU mortality, and in-hospital mortality. Twenty-eight-day ICU mortality was defined as death in the ICU within 28 days of study day 1, overall ICU mortality as death at any time during the ICU stay, and in-hospital mortality as death during the index hospitalization. Secondary outcomes included survival time, changes over time in APACHE II and SOFA scores, and organ support requirements. Primary mortality outcomes and survival analyses were also performed in the septic shock subgroup and according to ICU admission type. Post hoc subgroup analyses were performed according to IEAT appropriateness.

### 2.5. Statistical Analysis

Continuous variables were assessed for normality using the Shapiro–Wilk test. Normally distributed variables were expressed as mean ± standard deviation and compared using the independent-samples Student’s *t*-test. Non-normally distributed variables were expressed as median (minimum–maximum) and compared using the Mann–Whitney U test. Categorical variables were summarized as counts and percentages and compared using Pearson’s chi-square test or Fisher’s exact test, as appropriate.

Mortality outcomes were analyzed as binary variables. Relative risks (RRs) and risk differences were calculated from observed event proportions, with corresponding 95% confidence intervals (CIs). Risk differences were calculated as the control-group event proportion minus the IgGAM-group event proportion, such that positive values indicate lower mortality in the IgGAM group. Given the small sample size, limited number of outcome events, and sparse event distributions within the post hoc IEAT subgroups, multivariable regression was not performed.

Kaplan–Meier curves were constructed for time to death, and survival distributions were compared using the log-rank test. Survival time was calculated from study day 1 to death or the last documented follow-up. Dates of death and vital status during follow-up were ascertained from hospital records, including subsequent hospital admissions. Patients who remained alive at their last documented follow-up were right-censored at that time. The number and timing of censoring events are reported in Supplementary Table S6. Restricted mean survival time (RMST) was calculated as the area under the Kaplan–Meier survival curve through a common restriction time (τ) of 60 days. This restriction time was selected because follow-up extended beyond 60 days in both treatment groups across the overall cohort and all subgroup analyses, avoiding extrapolation beyond the observed follow-up. RMST differences were calculated as IgGAM minus control, with positive values indicating longer restricted survival time in the IgGAM group. The Greenwood plug-in estimator was used for the asymptotic variance of RMST estimates. Between-group RMST differences are presented with 95% CIs; separate *p* values for RMST differences are not reported. Log-rank test statistics are reported as χ^2^ values with 1 degree of freedom.

Longitudinal APACHE II and SOFA analyses were restricted to patients with measurements available at all seven prespecified study days. Changes over time within each treatment group were assessed using the Friedman test, followed by Bonferroni-corrected post hoc pairwise comparisons. Descriptive statistics and between-group comparisons at individual study days in the longitudinal analyses were based on the same complete-case populations. Study day 1 baseline comparisons in the overall cohort were based on the full overall cohort, whereas those reported in Supplementary Table S5 for the IEAT subgroups were based on the full IEAT subgroups. No direct between-group comparison of longitudinal change was performed. Except for the Bonferroni correction applied to post hoc pairwise comparisons following the Friedman test, no adjustment for multiple testing was applied to between-group comparisons across individual study days, outcomes, or subgroup analyses. All tests were two-sided, and a *p* value < 0.05 was considered statistically significant. All analyses other than the RMST analyses were performed using IBM SPSS Statistics for Windows, version 26.0 (IBM Corp., Armonk, NY, USA). RMST analyses were performed using R version 4.6.1 (R Foundation for Statistical Computing, Vienna, Austria) with the survRM2 package (version 1.0-4).

## 3. Results

### 3.1. Demographic and Clinical Characteristics at Study Inclusion

Patients in the IgGAM group were younger than those in the control group (47.4 ± 21 vs. 60.7 ± 18 years; *p* = 0.034). Other demographic characteristics, ICU admission characteristics, and comorbidities presented in Table 1 did not differ significantly between groups.

In the full cohort, on study day 1, both the APACHE II score [15 (5–25) vs. 18 (12–37); *p* = 0.026] and the SOFA score [7 (2–18) vs. 10 (3–15); *p* = 0.034] were lower in the IgGAM group than in the control group. Sepsis stage did not differ significantly between groups, and septic shock was the predominant clinical presentation in both groups. Vasopressor therapy on study day 1 was required in 15 patients (60%) in the IgGAM group and 19 patients (76%) in the control group (*p* = 0.225). No significant between-group differences were observed in MAP, CVP, laboratory parameters, or ABG values (Table 2).

Appropriate IEAT was less frequent in the IgGAM group than in the control group (12/25 [48%] vs. 19/25 [76%]; *p* = 0.041). No significant between-group differences were observed in individual infection sites. Pulmonary infection was the most common site in both groups. The second most common infection site was intra-abdominal infection in the IgGAM group and catheter-related bacteremia in the control group. All patients were culture-positive, and hospital-acquired infections predominated in both groups (Table 3). No significant between-group differences were observed in the broad microorganism categories (Supplementary Table S1). Baseline characteristics by treatment group according to IEAT appropriateness are presented in Supplementary Table S5.

### 3.2. Mortality and Survival

**Figure 1:**
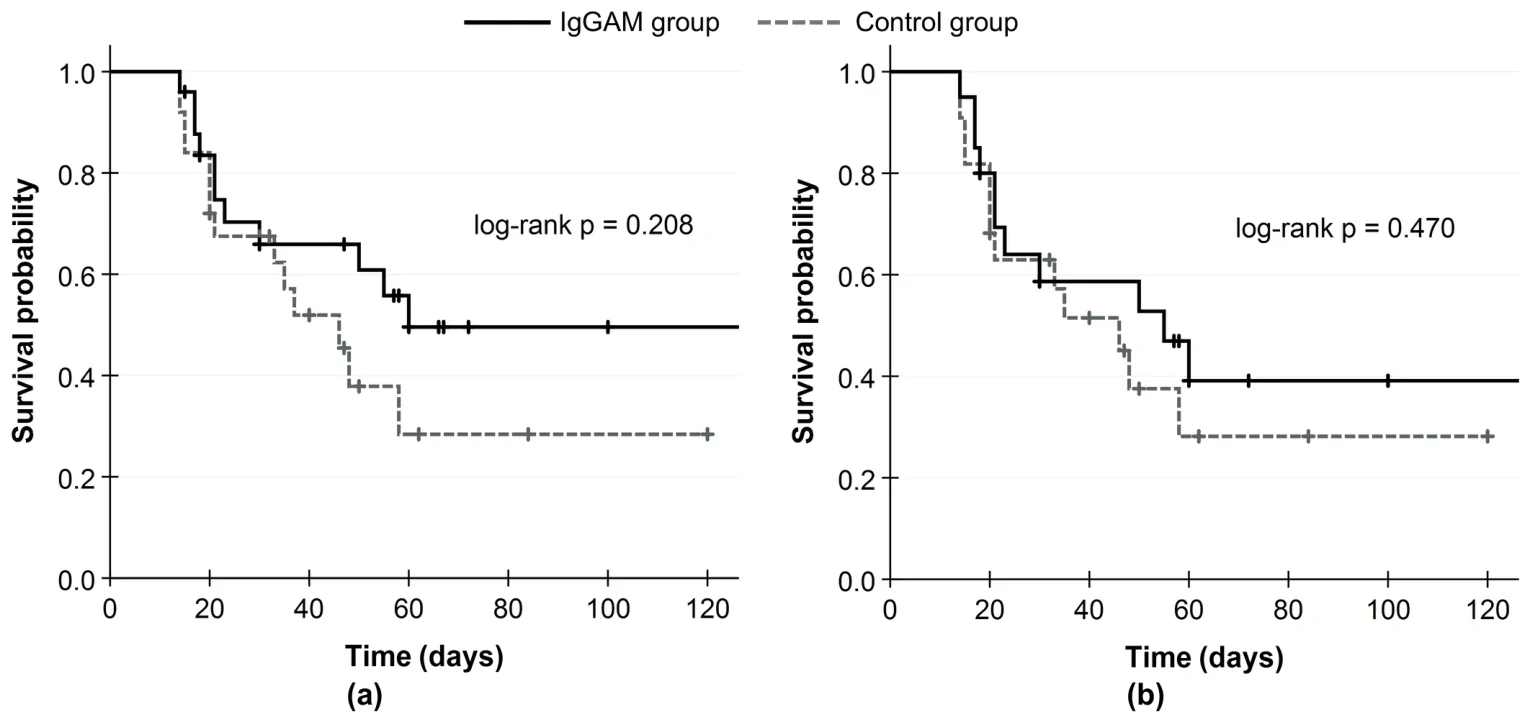
Kaplan–Meier survival curves for time to death in (**a**) the overall cohort (*n* = 50; IgGAM group, *n* = 25; control group, *n* = 25) and (**b**) patients with septic shock (*n* = 42; IgGAM group, *n* = 20; control group, *n* = 22). Survival distributions did not differ significantly between groups in either analysis (overall cohort, log-rank χ^2^ = 1.588, *p* = 0.208; septic shock subgroup, log-rank χ^2^ = 0.523, *p* = 0.470). Censoring is indicated by plus signs (+).

**Figure 2:**
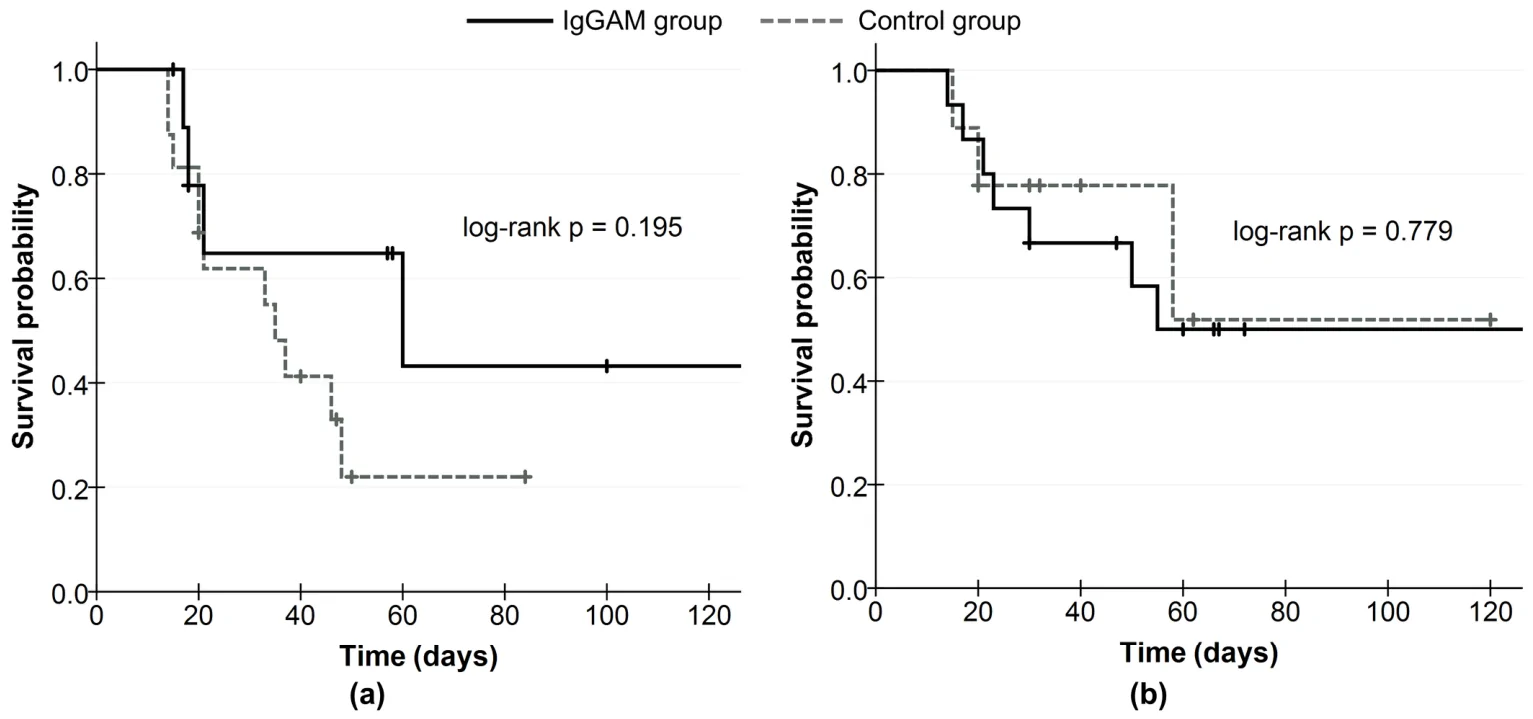
Kaplan–Meier survival curves for time to death according to ICU admission type: (**a**) medical admission (*n* = 26; IgGAM group, *n* = 10; control group, *n* = 16) and (**b**) surgical admission (*n* = 24; IgGAM group, *n* = 15; control group, *n* = 9). Survival distributions did not differ significantly between groups in either subgroup (medical admission, log-rank χ^2^ = 1.680, *p* = 0.195; surgical admission, log-rank χ^2^ = 0.078, *p* = 0.779). Censoring is indicated by plus signs (+).

**Figure 3:**
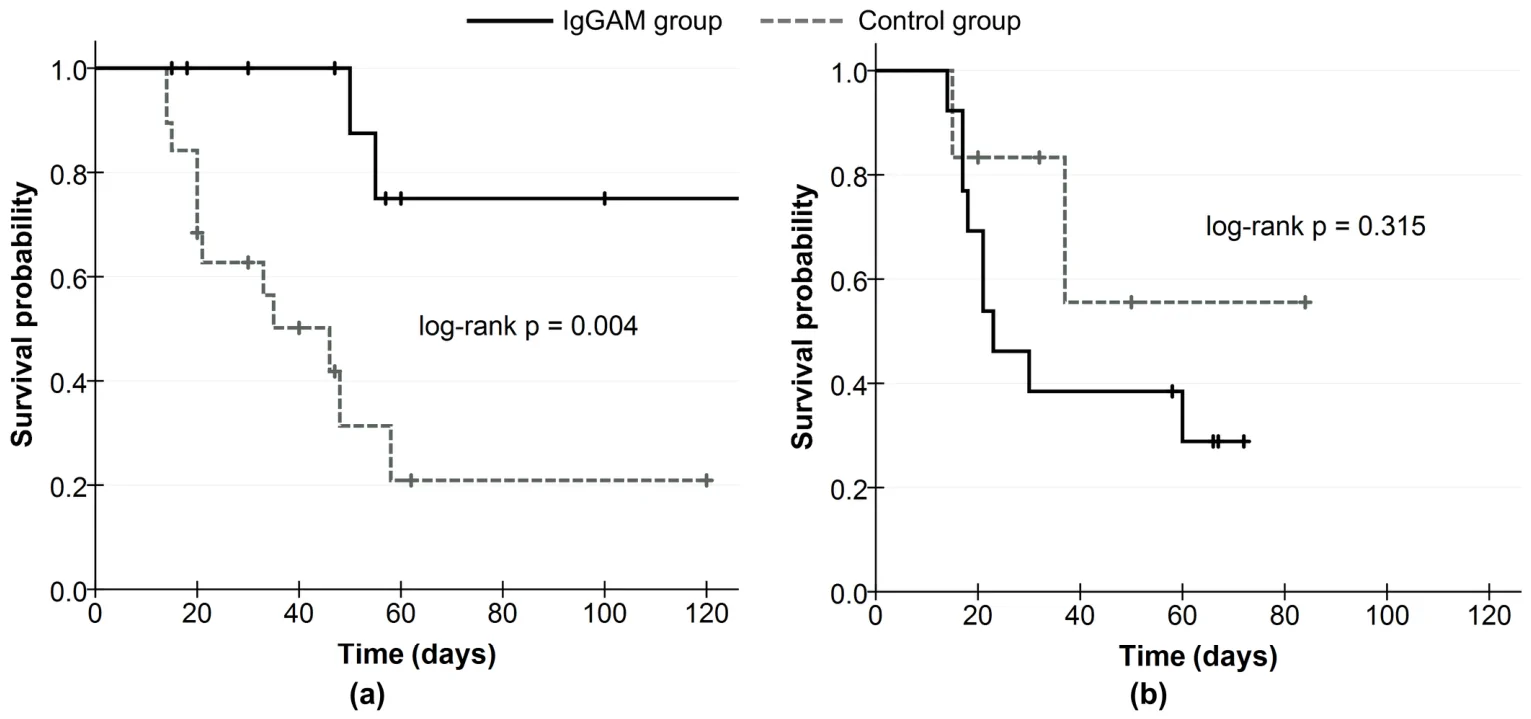
Kaplan–Meier survival curves for time to death in the post hoc subgroups according to initial empiric antibiotic therapy (IEAT) appropriateness: (**a**) appropriate IEAT (*n* = 31; IgGAM group, *n* = 12; control group, *n* = 19) and (**b**) inappropriate IEAT (*n* = 19; IgGAM group, *n* = 13; control group, *n* = 6). Survival distributions differed significantly between groups in the subgroup receiving appropriate IEAT (log-rank χ^2^ = 8.076, *p* = 0.004), but did not differ significantly in the subgroup receiving inappropriate IEAT (log-rank χ^2^ = 1.008, *p* = 0.315). Censoring is indicated by plus signs (+).

Mortality outcomes and risk differences are summarized in Table 4, and 60-day restricted mean survival time (RMST) estimates and between-group RMST differences are summarized in Table 5. Kaplan–Meier survival curves are shown in Figure 1, Figure 2 and Figure 3. Observed follow-up ranged from 14 to 260 days; group- and subgroup-specific follow-up ranges and censoring details are provided in Supplementary Table S6.

In the overall cohort (*n* = 50), 28-day ICU mortality was 28.0% in the IgGAM group and 32.0% in the control group (RR, 0.88; 95% CI, 0.37–2.05; *p* = 0.758). Overall ICU mortality was 48.0% in the IgGAM group and 52.0% in the control group (RR, 0.92; 95% CI, 0.53–1.61; *p* = 0.777), and in-hospital mortality was 48.0% and 56.0% (RR, 0.86; 95% CI, 0.50–1.46; *p* = 0.571). The Kaplan–Meier survival distributions did not differ significantly between groups (log-rank χ^2^ = 1.588; *p* = 0.208; Figure 1a). The 60-day RMST was 45.7 ± 3.9 days in the IgGAM group and 40.3 ± 3.8 days in the control group, corresponding to an RMST difference of 5.4 days (95% CI, −5.2 to 16.0; Table 5).

In patients with septic shock (*n* = 42), 28-day ICU mortality was 35.0% in the IgGAM group and 36.4% in the control group (RR, 0.96; 95% CI, 0.43–2.17; *p* = 0.927). Overall ICU mortality was 60.0% in the IgGAM group and 54.5% in the control group (RR, 1.10; 95% CI, 0.65–1.86; *p* = 0.721), and in-hospital mortality was 60.0% and 59.1% (RR, 1.02; 95% CI, 0.62–1.67; *p* = 0.952). The Kaplan–Meier survival distributions did not differ significantly between groups (log-rank χ^2^ = 0.523; *p* = 0.470; Figure 1b). The 60-day RMST was 42.7 ± 4.4 days in the IgGAM group and 39.3 ± 4.1 days in the control group, corresponding to an RMST difference of 3.4 days (95% CI, −8.4 to 15.2; Table 5).

In patients admitted to the ICU for medical reasons (*n* = 26), 28-day ICU mortality was 30.0% in the IgGAM group and 37.5% in the control group (RR, 0.80; 95% CI, 0.26–2.50; *p* = 1.000). Overall ICU mortality and in-hospital mortality were 40.0% in the IgGAM group and 68.8% in the control group (RR, 0.58; 95% CI, 0.25–1.33; *p* = 0.228). The Kaplan–Meier survival distributions did not differ significantly between groups (log-rank χ^2^ = 1.680; *p* = 0.195; Figure 2a). The 60-day RMST was 45.5 ± 6.8 days in the IgGAM group and 36.1 ± 4.4 days in the control group, corresponding to an RMST difference of 9.4 days (95% CI, −6.5 to 25.2; Table 5).

In patients admitted to the ICU for surgical reasons (*n* = 24), 28-day ICU mortality was 26.7% in the IgGAM group and 22.2% in the control group (RR, 1.20; 95% CI, 0.27–5.29; *p* = 1.000). Overall ICU mortality was 53.3% in the IgGAM group and 22.2% in the control group (RR, 2.40; 95% CI, 0.65–8.90; *p* = 0.210), and in-hospital mortality was 53.3% and 33.3% (RR, 1.60; 95% CI, 0.57–4.52; *p* = 0.423). The Kaplan–Meier survival distributions did not differ significantly between groups (log-rank χ^2^ = 0.078; *p* = 0.779; Figure 2b). The 60-day RMST was 45.8 ± 4.7 days in the IgGAM group and 50.0 ± 5.8 days in the control group, corresponding to an RMST difference of −4.3 days (95% CI, −18.9 to 10.3; Table 5).

In the post hoc subgroup receiving appropriate IEAT (*n* = 31), 28-day ICU mortality was 0.0% in the IgGAM group (0/12) and 36.8% in the control group (7/19) (risk difference, 36.8 percentage points; 95% CI, 15.2–58.5; *p* = 0.026). Overall ICU mortality was 25.0% in the IgGAM group and 57.9% in the control group (RR, 0.43; 95% CI, 0.15–1.24; *p* = 0.073). In-hospital mortality was 25.0% in the IgGAM group and 63.2% in the control group (risk difference, 38.2 percentage points; 95% CI, 5.4–70.9; *p* = 0.038). Kaplan–Meier survival distributions differed significantly between groups (log-rank χ^2^ = 8.076; *p* = 0.004; Figure 3a). The 60-day RMST was 58.1 ± 1.2 days in the IgGAM group and 38.4 ± 4.3 days in the control group, corresponding to an RMST difference of 19.8 days (95% CI, 11.1 to 28.4; Table 5).

In the post hoc subgroup receiving inappropriate IEAT (*n* = 19), 28-day ICU mortality was 53.8% in the IgGAM group and 16.7% in the control group (RR, 3.23; 95% CI, 0.50–20.73; *p* = 0.177). Overall ICU mortality and in-hospital mortality were 69.2% in the IgGAM group and 33.3% in the control group (RR, 2.08; 95% CI, 0.63–6.81; *p* = 0.319). The Kaplan–Meier survival distributions did not differ significantly between groups (log-rank χ^2^ = 1.008; *p* = 0.315; Figure 3b). The 60-day RMST was 35.5 ± 5.5 days in the IgGAM group and 46.1 ± 7.7 days in the control group, corresponding to an RMST difference of −10.6 days (95% CI, −29.2 to 7.9; Table 5).

### 3.3. APACHE II and SOFA Scores over Time

**Figure 4:**
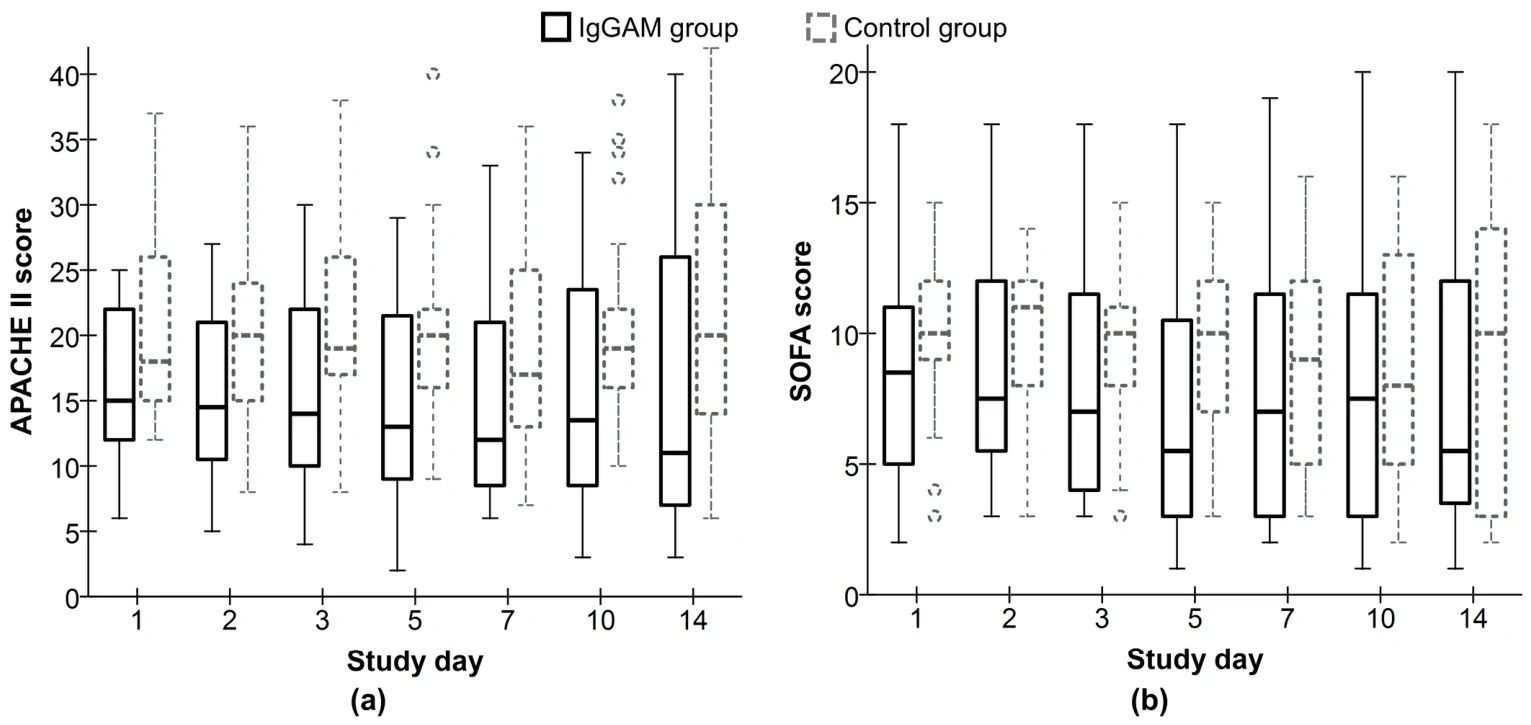
Distribution of (**a**) APACHE II scores and (**b**) SOFA scores over time by treatment group on study days 1, 2, 3, 5, 7, 10, and 14 in the complete-case longitudinal analysis of the overall cohort (IgGAM group, *n* = 20; control group, *n* = 25). Boxes extend from the 25th to the 75th percentile; horizontal lines within the boxes indicate medians; whiskers extend to the minimum and maximum non-outlier values; circles indicate outliers.

Full-cohort study day 1 APACHE II and SOFA comparisons are reported in Table 2. In the complete-case longitudinal APACHE II analysis, which included 20 patients in the IgGAM group and 25 patients in the control group, median APACHE II scores were lower in the IgGAM group than in the control group on study days 1, 3, 5, and 14. APACHE II scores did not change significantly over time within either group (Supplementary Table S2, Figure 4a).

In the complete-case longitudinal SOFA analysis, which included 20 patients in the IgGAM group and 25 patients in the control group, median SOFA scores decreased significantly over time in the IgGAM group (*p* = 0.017) and were lower on study days 5 and 14 than on study day 1. No significant within-group change was observed in the control group (*p* = 0.616). Between-group differences in SOFA scores did not reach statistical significance at any study day within this complete-case population (Supplementary Table S3, Figure 4b).

**Figure 5:**
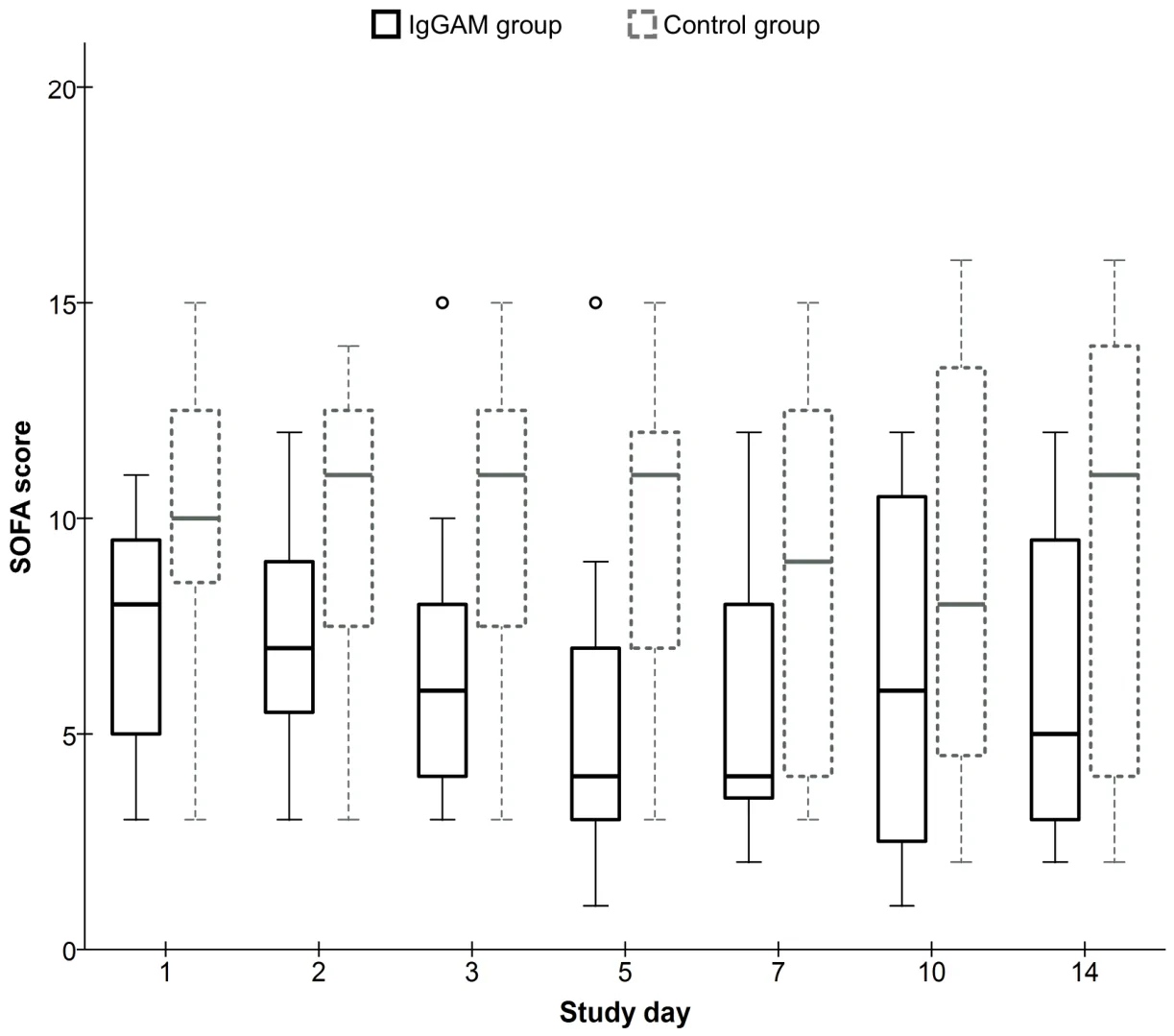
Distribution of SOFA scores over time by treatment group on study days 1, 2, 3, 5, 7, 10, and 14 in the complete-case longitudinal analysis of the post hoc subgroup receiving appropriate initial empiric antibiotic therapy (IEAT) (IgGAM group, *n* = 11; control group, *n* = 19). Boxes extend from the 25th to the 75th percentile; horizontal lines within the boxes indicate medians; whiskers extend to the minimum and maximum non-outlier values; circles indicate outliers.

In the full post hoc subgroup receiving appropriate IEAT, study day 1 APACHE II and SOFA scores were lower in the IgGAM group than in the control group [APACHE II, 12.5 (5–23) vs. 18 (12–37), *p* = 0.004; SOFA, 7.5 (3–11) vs. 10 (3–15), *p* = 0.015; Supplementary Table S5]. In the complete-case longitudinal SOFA analysis of this subgroup, which included 11 patients in the IgGAM group and 19 patients in the control group, SOFA scores decreased significantly over time in the IgGAM group (*p* = 0.006) and were lower on study days 5 and 7 than on study day 1. No significant within-group change was observed in the control group (*p* = 0.443). Median SOFA scores were lower in the IgGAM group than in the control group on study days 1, 2, 3, and 5 (Supplementary Table S4, Figure 5).

### 3.4. Organ Support and Supportive Therapies During ICU Stay

In the overall cohort, vasopressor therapy during the ICU stay was required less frequently in the IgGAM group than in the control group (68% vs. 92%; *p* = 0.034). The duration of vasopressor therapy did not differ significantly between groups. All patients in both groups required IMV, and the duration of IMV did not differ significantly between groups.

The use of individual RRT modalities did not differ significantly between groups. The use of TPE, intravenous hydrocortisone for refractory shock, and LMWH prophylaxis also did not differ significantly between groups (Table 6).

## 4. Discussion

In this retrospective analysis of ICU patients with sepsis or septic shock, adjunctive IgGAM therapy was not associated with significant differences in 28-day ICU mortality, overall ICU mortality, or in-hospital mortality, and Kaplan–Meier survival distributions did not differ significantly between groups in the overall cohort. The 60-day RMST was 5.4 days longer in the IgGAM group, although the 95% CI for the between-group difference included zero (−5.2 to 16.0 days). Baseline APACHE II and SOFA scores were lower in the IgGAM group, which may have favored outcomes. Vasopressor use during the ICU stay was less frequent in the IgGAM group; however, a lower proportion of patients in this group also required vasopressors on study day 1, although this baseline difference was not statistically significant. In the complete-case longitudinal SOFA analysis, SOFA scores decreased significantly over time within the IgGAM group. As longitudinal changes were not directly compared between groups, these findings do not establish a differential treatment effect on organ dysfunction.

In the post hoc subgroup receiving appropriate IEAT, adjunctive IgGAM therapy, compared with standard sepsis management alone, was associated with lower 28-day ICU mortality, lower in-hospital mortality, and a 19.8-day longer 60-day RMST (95% CI, 11.1–28.4 days). Kaplan–Meier survival distributions also differed significantly between groups. Baseline APACHE II and SOFA scores were lower in the IgGAM group, which may have favored outcomes in this subgroup. In the complete-case longitudinal SOFA analysis, SOFA scores decreased significantly over time in the IgGAM group, whereas no significant within-group change was observed in the control group. Interpretation of this longitudinal pattern is further limited by the complete-case restriction and the absence of a direct between-group comparison of change over time. The observed differences in mortality risk should be interpreted cautiously given the small subgroup size, the post hoc nature of the analysis, and the baseline differences in APACHE II and SOFA scores. By contrast, in the subgroup receiving inappropriate IEAT, point estimates for 28-day ICU mortality, overall ICU mortality, and in-hospital mortality were higher in the IgGAM group, although none of these differences reached statistical significance.

The association observed in the appropriate-IEAT subgroup is consistent with findings from a small randomized controlled trial in surgical patients with intra-abdominal severe sepsis. In that study, inappropriate initial antibiotic therapy was the only variable independently associated with death (OR, 19.4; 95% CI, 1.17–324.1), and among evaluable patients receiving adequate initial antibiotic therapy, mortality was lower with adjunctive IgM-enriched IVIG than with control treatment (8.7% vs. 33.3%) [26]. Earlier observational and meta-analytic studies have linked inappropriate empiric antimicrobial therapy with higher mortality in sepsis, while delays in effective antimicrobial administration have also been associated with worse outcomes, particularly in septic shock [27,28,29,30]. More recent meta-analytic evidence in adults with bacteremia similarly found an association between inappropriate empiric antimicrobial therapy and higher mortality, including in patients with severe sepsis or septic shock [31]. A separate systematic review and meta-analysis found that antibiotic administration within 3 h, compared with later administration, was associated with lower mortality in patients with sepsis [32]. Taken together, these findings support considering both antimicrobial appropriateness and timing in prospective evaluations of adjunctive IgGAM therapy.

In subgroup analyses according to ICU admission type, no statistically significant differences in mortality were observed between the IgGAM and control groups. Mortality point estimates were lower in the IgGAM group than in the control group in the medical-admission subgroup, whereas the opposite pattern was observed in the surgical-admission subgroup. A 2023 single-center retrospective analysis of 44 adults with sepsis or septic shock after major abdominal surgery found no significant difference in mortality between patients receiving pentameric IgM plus antibiotics and those receiving antibiotics alone [33]. By contrast, a recent single-center retrospective study of 108 adult surgical patients with sepsis or septic shock reported that IgM-enriched immunoglobulin therapy was associated with lower ICU and in-hospital mortality after inverse probability weighting with regression adjustment [34]. In that study, empiric antibiotic appropriateness was reported to be comparable between treatment groups, although residual confounding could not be excluded because of the observational design [34]. Given the importance of timely and adequate source control in sepsis management [35], the differing findings across studies may reflect differences in patient selection, sepsis etiology, illness severity, antimicrobial management, treatment selection, and source-control characteristics. However, source-control data in the present study were insufficient to evaluate these possibilities.

Evidence regarding the effects of standard IgG-only IVIG and IgM/IgA-enriched immunoglobulin preparations on mortality in sepsis remains conflicting. A 2007 meta-analysis of 15 adult trials reported lower mortality with polyvalent immunoglobulins overall (RR, 0.79; 95% CI, 0.69–0.90); subgroup estimates were more favorable for IgGAM (RR, 0.66; 95% CI, 0.51–0.84) than for IgG-only preparations (RR, 0.85; 95% CI, 0.73–0.99), although these estimates did not represent a direct head-to-head comparison between formulations [36]. Turgeon et al. similarly reported lower mortality with polyclonal IVIG compared with placebo or no intervention (RR, 0.74; 95% CI, 0.62–0.89) [15]. Laupland et al. also found an overall reduction in mortality with polyclonal IVIG (OR, 0.66; 95% CI, 0.53–0.83); however, this association was not observed when the analysis was restricted to high-quality studies (OR, 0.96; 95% CI, 0.71–1.30) [16]. In contrast, the phase III multicenter SBITS trial of standard IgG-only IVIG in patients with score-defined severe sepsis found no reduction in 28-day mortality (37.3% with placebo vs. 39.3% with IVIG; *p* = 0.6695) [17].

Subsequent systematic reviews and meta-analyses have continued to produce mixed results. In the 2013 Cochrane review, sensitivity analysis restricted to five adult trials at low risk of bias, three evaluating standard polyclonal IVIG and two evaluating IgM-enriched IVIG, showed no significant reduction in mortality (RR, 0.97; 95% CI, 0.81–1.15) [37]. A subsequent meta-analysis of 18 RCTs reported lower mortality with IVIG overall (OR, 0.50; 95% CI, 0.34–0.71), but the authors emphasized the low methodological quality of many included studies, heterogeneity in dosing regimens and immunoglobulin preparations, and differences in control interventions [38]. Treatment effects also tended to be smaller or less consistent in studies meeting individual methodological quality criteria [38]. Pan et al. reported an overall reduction in mortality across 31 RCTs (RR, 0.86; 95% CI, 0.77–0.95); in formulation-specific analyses, the association was significant for IgM-enriched IVIG (RR, 0.55; 95% CI, 0.40–0.76) but not for standard IVIG (RR, 0.91; 95% CI, 0.81–1.02) [39]. More recently, a systematic review and meta-analysis restricted its core synthesis to randomized adult Pentaglobin trials and found a directionally favorable but statistically inconclusive association with short-term mortality (OR, 0.65; 95% CI, 0.39–1.07); the analysis restricted to exact 28-day mortality was likewise nonsignificant (OR, 0.84; 95% CI, 0.50–1.43) [40]. The review emphasized that IgG-only and IgM/IgA-enriched preparations should not be considered biologically or clinically interchangeable and concluded that current randomized evidence does not support routine use of IgM-enriched immunoglobulin in unselected adults with sepsis or septic shock, while supporting further adequately powered, phenotype-guided trials [40].

In the present study, appropriate IEAT was significantly less frequent in the IgGAM group, which may have influenced the overall mortality and survival comparisons. The IgGAM group also included a higher proportion of surgical admissions, although the difference in admission type was not statistically significant. Mortality point estimates differed in direction across admission-type subgroups, but none of these subgroup differences reached statistical significance. Given the marked heterogeneity of sepsis, variation in patient characteristics, illness severity, infection sites, antimicrobial appropriateness, treatment selection, and source-control requirements complicates assessment of the independent contribution of adjunctive IgGAM therapy.

Strengths of this study include the detailed microbiological characterization of the cohort, assessment of IEAT appropriateness, serial assessment of APACHE II and SOFA scores at prespecified study days, and complementary Kaplan–Meier and RMST analyses. Together, these features provide broader clinical context for interpreting the mortality and survival findings in relation to antimicrobial appropriateness and patterns of organ dysfunction over time. The findings do not support routine IgGAM use. Rather, they provide a rationale for prospective evaluation of adjunctive IgGAM therapy within optimized sepsis care, particularly when appropriate IEAT and, when required, adequate source control are ensured. Future studies should prespecify analyses according to IEAT appropriateness, systematically document the timing and adequacy of source control, and be adequately powered to evaluate treatment-by-IEAT interactions.

### Limitations

This study has several limitations. Its retrospective, nonrandomized design and clinician-directed selection of patients for IgGAM treatment introduce the potential for selection bias and confounding by indication. The single-center setting may limit generalizability, and the small sample size (*n* = 50; 25 patients per treatment group) limited statistical power, particularly in the post hoc IEAT subgroups (*n* = 31 with appropriate IEAT and *n* = 19 with inappropriate IEAT). Baseline imbalances may also have influenced between-group comparisons. In the overall cohort, younger age and lower baseline APACHE II and SOFA scores in the IgGAM group may have favored outcomes, whereas the lower frequency of appropriate IEAT may have been associated with less favorable outcomes. In the appropriate-IEAT subgroup, lower baseline APACHE II and SOFA scores in the IgGAM group may likewise have favored outcomes. Potential confounding by major baseline prognostic factors could not be addressed through reliable multivariable adjustment, and residual confounding by measured and unmeasured factors cannot be excluded. The longitudinal APACHE II and SOFA analyses were restricted to patients with complete measurements across all seven prespecified study days. This complete-case approach reduced the sample available for longitudinal analyses, particularly in the IgGAM group, and may have introduced attrition bias if the availability of serial measurements was related to clinical course or outcome.

The analyses according to IEAT appropriateness were post hoc, and no treatment-by-IEAT interaction analysis was performed. Therefore, these subgroup findings should be considered hypothesis-generating and should not be interpreted as evidence that IEAT appropriateness modifies the association of IgGAM therapy with outcomes. The number of statistical comparisons across outcomes and subgroup analyses may also have increased the risk of type I error. Available data on the adequacy and timing of source control were insufficient for reliable stratified analyses, particularly in surgical patients. Residual confounding related to source control therefore cannot be excluded.

## 5. Conclusions

Adjunctive IgGAM therapy was not associated with significant differences in mortality in the overall cohort. However, in the post hoc subgroup receiving appropriate IEAT, IgGAM therapy was associated with lower 28-day ICU and in-hospital mortality and a longer 60-day RMST. Given the potential for residual confounding and selection bias and the number of statistical comparisons performed, these subgroup findings should be considered hypothesis-generating. Prospective studies are warranted to evaluate adjunctive IgGAM therapy within a clinical framework in which appropriate IEAT and adequate source control are ensured.

## Figures and Tables

**Table 1:** Demographic and clinical characteristics at study inclusion.

Characteristic	IgGAM Group (*n* = 25)	Control Group (*n* = 25)	*p* Value
**Demographics**			
Age, years, mean ± SD	47.4 ± 21	60.7 ± 18	0.034
Body weight, kg, mean ± SD	79.3 ± 16	73.4 ± 10	0.235
Sex, *n* (%)			0.152
Female	13 (52)	8 (32)	
Male	12 (48)	17 (68)	
**ICU admission type, *n* (%)**			0.162
Medical	10 (40)	16 (64)	
Surgical	15 (60)	9 (36)	
**Admission source, *n* (%)**			0.758
Same hospital	17 (68)	18 (72)	
External hospital	8 (32)	7 (28)	
**Pre-ICU location, *n* (%)**			0.288
Ward	8 (32)	7 (28)	
Another ICU	9 (36)	14 (56)	
Operating room	8 (32)	4 (16)	
**Comorbidities, *n* (%)**			
Hypertension	6 (24)	12 (48)	0.077
Coronary artery disease	5 (20)	9 (36)	0.208
Congestive heart failure	5 (20)	8 (32)	0.333
Chronic obstructive pulmonary disease	4 (16)	0 (0)	0.110
Diabetes mellitus	7 (28)	5 (20)	0.508
Chronic kidney disease	0 (0)	3 (12)	0.235
Chronic liver disease	3 (12)	1 (4)	0.609

Abbreviations: ICU, intensive care unit; IgGAM, IgM- and IgA-enriched polyclonal intravenous immunoglobulin; SD, standard deviation.

**Table 2:** Sepsis stage, clinical parameters, laboratory findings, and arterial blood gas values on study day 1.

Parameter	IgGAM Group (*n* = 25)	Control Group (*n* = 25)	*p* Value
**Sepsis stage**			0.702
Sepsis, *n* (%)	5 (20)	3 (12)	
Septic shock, *n* (%)	20 (80)	22 (88)	
APACHE II score, median (min–max)	15 (5–25)	18 (12–37)	0.026
SOFA score, median (min–max)	7 (2–18)	10 (3–15)	0.034
**Vasopressor therapy, *n* (%)**	15 (60)	19 (76)	0.225
**Hemodynamic parameters, median (min–max)**			
Mean arterial pressure, mmHg	63 (50–97)	63 (46–138)	0.969
Central venous pressure, mmHg	7 (4–18)	8 (3–16)	0.565
**Laboratory parameters, median (min–max)**			
White blood cell count, 10^3^/µL	11 (0.3–33.5)	11 (0.1–35.1)	0.977
Platelet count, 10^3^/µL	179 (11–1090)	152 (5–442)	0.628
Creatinine, mg/dL	0.9 (0.4–4.5)	0.9 (0.2–3.9)	0.869
Total bilirubin, mg/dL	1.1 (0.3–19)	0.8 (0.3–14.4)	0.712
C-reactive protein, mg/L	92 (13–310)	125 (24–400)	0.182
Procalcitonin, ng/mL	9.5 (0.2–63)	4.2 (0.3–180)	0.443
INR	1.2 (0.8–2.8)	1.4 (1.1–2.3)	0.078
aPTT, s	31 (21–66)	35 (22–55)	0.854
**Arterial blood gas values, median (min–max)**			
PaO_2_/FiO_2_ ratio, mmHg	190 (54–357)	210 (66–526)	0.669
Lactate, mmol/L	2.3 (0.4–19)	2.2 (1.3–34)	0.503
Bicarbonate, mmol/L	22.0 (15–34)	21.8 (14–36)	0.763
Base deficit, mEq/L	−3.0 (−10 to 12)	−3.5 (−14 to 13)	0.432

Abbreviations: APACHE II, Acute Physiology and Chronic Health Evaluation II; aPTT, activated partial thromboplastin time; IgGAM, IgM- and IgA-enriched polyclonal intravenous immunoglobulin; INR, international normalized ratio; PaO_2_/FiO_2_, ratio of arterial partial pressure of oxygen to fraction of inspired oxygen; SOFA, Sequential Organ Failure Assessment.

**Table 3:** Infection characteristics and appropriateness of initial empiric antibiotic therapy.

Variable	IgGAM Group (*n* = 25)	Control Group (*n* = 25)	*p* Value
Infection sites *, *n* (%)			
Pulmonary	23 (92)	21 (84)	0.667
Intra-abdominal	14 (56)	10 (40)	0.258
Urinary	10 (40)	15 (60)	0.157
Catheter-related bacteremia	11 (44)	17 (68)	0.087
Primary bacteremia	0 (0)	2 (8)	0.490
**Culture status, *n* (%)**			–
Culture-positive	25 (100)	25 (100)	
Culture-negative	0 (0)	0 (0)	
**Acquisition type, *n* (%)**			1.000
Hospital-acquired	20 (80)	21 (84)	
Community-acquired	5 (20)	4 (16)	
**IEAT appropriateness, *n* (%)**			0.041
Appropriate IEAT	12 (48)	19 (76)	
Inappropriate IEAT	13 (52)	6 (24)	

* More than one infection site could be identified in the same patient; therefore, percentages for infection sites may sum to more than 100%. Abbreviations: IEAT, initial empiric antibiotic therapy; IgGAM, IgM- and IgA-enriched polyclonal intravenous immunoglobulin.

**Table 4:** Mortality outcomes in the overall cohort and subgroups.

Cohort or Subgroup (*n*; IgGAM/Control)	IgGAM Group	Control Group	Risk Difference * (Control − IgGAM) Percentage Points [95% CI]	*p* Value
**Overall cohort** (*n* = 50; 25/25)				
28-day ICU mortality	28.0% (7/25)	32.0% (8/25)	4.0 [−21.4 to 29.4]	0.758
Overall ICU mortality	48.0% (12/25)	52.0% (13/25)	4.0 [−23.7 to 31.7]	0.777
In-hospital mortality	48.0% (12/25)	56.0% (14/25)	8.0 [−19.6 to 35.6]	0.571
**Septic shock** (*n* = 42; 20/22)				
28-day ICU mortality	35.0% (7/20)	36.4% (8/22)	1.4 [−27.6 to 30.4]	0.927
Overall ICU mortality	60.0% (12/20)	54.5% (12/22)	−5.5 [−35.4 to 24.4]	0.721
In-hospital mortality	60.0% (12/20)	59.1% (13/22)	−0.9 [−30.6 to 28.8]	0.952
**Medical admission** (*n* = 26; 10/16)				
28-day ICU mortality	30.0% (3/10)	37.5% (6/16)	7.5 [−29.5 to 44.5]	1.000
Overall ICU mortality	40.0% (4/10)	68.8% (11/16)	28.8 [−9.2 to 66.7]	0.228
In-hospital mortality	40.0% (4/10)	68.8% (11/16)	28.8 [−9.2 to 66.7]	0.228
**Surgical admission** (*n* = 24; 15/9)				
28-day ICU mortality	26.7% (4/15)	22.2% (2/9)	−4.4 [−39.6 to 30.7]	1.000
Overall ICU mortality	53.3% (8/15)	22.2% (2/9)	−31.1 [−68.2 to 6.0]	0.210
In-hospital mortality	53.3% (8/15)	33.3% (3/9)	−20.0 [−59.8 to 19.8]	0.423
Appropriate IEAT ** (*n* = 31; 12/19)				
28-day ICU mortality	0.0% (0/12)	36.8% (7/19)	36.8 [15.2 to 58.5]	0.026
Overall ICU mortality	25.0% (3/12)	57.9% (11/19)	32.9 [−0.2 to 66.0]	0.073
In-hospital mortality	25.0% (3/12)	63.2% (12/19)	38.2 [5.4 to 70.9]	0.038
Inappropriate IEAT ** (*n* = 19; 13/6)				
28-day ICU mortality	53.8% (7/13)	16.7% (1/6)	−37.2 [−77.5 to 3.1]	0.177
Overall ICU mortality	69.2% (9/13)	33.3% (2/6)	−35.9 [−81.2 to 9.4]	0.319
In-hospital mortality	69.2% (9/13)	33.3% (2/6)	−35.9 [−81.2 to 9.4]	0.319

* Risk differences were calculated as control minus IgGAM and are expressed as percentage points; positive values indicate lower mortality in the IgGAM group. ** Subgroup analyses according to IEAT appropriateness were performed post hoc. Abbreviations: CI, confidence interval; ICU, intensive care unit; IEAT, initial empiric antibiotic therapy; IgGAM, IgM- and IgA-enriched polyclonal intravenous immunoglobulin.

**Table 5:** Restricted mean survival time at 60 days in the overall cohort and subgroups.

Cohort or Subgroup (*n*; IgGAM/Control)	IgGAM Group RMST_60_ ± SE, Days	Control Group RMST_60_ ± SE, Days	RMST Difference *, Days (95% CI)	Log-Rank χ^2^	Log-Rank *p* Value
Overall cohort (*n* = 50; 25/25)	45.7 ± 3.9	40.3 ± 3.8	5.4 [−5.2 to 16.0]	1.588	0.208
Septic shock (*n* = 42; 20/22)	42.7 ± 4.4	39.3 ± 4.1	3.4 [−8.4 to 15.2]	0.523	0.470
Medical admission (*n* = 26; 10/16)	45.5 ± 6.8	36.1 ± 4.4	9.4 [−6.5 to 25.2]	1.680	0.195
Surgical admission (*n* = 24; 15/9)	45.8 ± 4.7	50.0 ± 5.8	−4.3 [−18.9 to 10.3]	0.078	0.779
Appropriate IEAT ** (*n* = 31; 12/19)	58.1 ± 1.2	38.4 ± 4.3	19.8 [11.1 to 28.4]	8.076	0.004
Inappropriate IEAT ** (*n* = 19; 13/6)	35.5 ± 5.5	46.1 ± 7.7	−10.6 [−29.2 to 7.9]	1.008	0.315

* RMST differences were calculated as IgGAM minus control; positive values indicate longer restricted survival time in the IgGAM group. RMST differences are presented with 95% confidence intervals; separate *p* values for RMST differences are not reported. Kaplan–Meier survival distributions over the available follow-up were compared using the log-rank test. All log-rank tests had 1 degree of freedom. ** Subgroup analyses according to IEAT appropriateness were performed post hoc. Abbreviations: CI, confidence interval; IEAT, initial empiric antibiotic therapy; IgGAM, IgM- and IgA-enriched polyclonal intravenous immunoglobulin; RMST, restricted mean survival time; SE, standard error.

**Table 6:** Organ support and supportive therapies during ICU stay.

Therapy *	IgGAM Group (*n* = 25)	Control Group (*n* = 25)	*p* Value
Vasopressor therapy, *n* (%)	17 (68)	23 (92)	0.034
Duration of vasopressor therapy, days, median (min–max)	12 (0–158)	10 (0–26)	0.690
IMV, *n* (%)	25 (100)	25 (100)	1.000
Duration of IMV, days, median (min–max)	17 (1–138)	20 (1–46)	0.930
Intermittent hemodialysis, *n* (%)	3 (12)	7 (28)	0.157
Continuous hemodialysis, *n* (%)	7 (28)	6 (24)	0.747
Continuous hemofiltration, *n* (%)	2 (8)	0 (0)	0.490
TPE, *n* (%)	16 (64)	11 (44)	0.156
Intravenous hydrocortisone for refractory shock, *n* (%)	12 (48)	11 (44)	0.777
LMWH prophylaxis, *n* (%)	24 (96)	24 (96)	1.000

* Categorical variables are presented as *n* (%), and duration variables are presented as median (minimum–maximum). For patients who did not receive vasopressor therapy, vasopressor duration was recorded as 0 days. Abbreviations: IgGAM, IgM- and IgA-enriched polyclonal intravenous immunoglobulin; IMV, invasive mechanical ventilation; LMWH, low-molecular-weight heparin; TPE, therapeutic plasma exchange.

## Data Availability

The de-identified data supporting the findings of this study are available from the corresponding author upon reasonable request, subject to applicable ethical and institutional requirements. The data are not publicly available due to privacy and ethical restrictions.

## Supplementary Materials

The following supporting information can be downloaded at: https://www.mdpi.com/article/10.3390/jcm15197731/s1, Figure S1: Analytical diagram of the study cohort and subgroup analyses; Table S1: Distribution of microorganisms isolated during the ICU stay; Table S2: APACHE II scores over time by treatment group in the complete-case longitudinal analysis of the overall cohort; Table S3: SOFA scores over time by treatment group in the complete-case longitudinal analysis of the overall cohort; Table S4: SOFA scores over time by treatment group in the complete-case longitudinal analysis of the post hoc subgroup receiving appropriate initial empiric antibiotic therapy; Table S5: Baseline characteristics by treatment group according to the appropriateness of initial empiric antibiotic therapy; Table S6: Follow-up duration and censoring in the Kaplan–Meier survival analyses.

## Abbreviations

The following abbreviations are used in this manuscript:

ABG	arterial blood gas
APACHE II	Acute Physiology and Chronic Health Evaluation II
aPTT	activated partial thromboplastin time
CI	confidence interval
CVP	central venous pressure
ICU	intensive care unit
IEAT	initial empiric antibiotic therapy
IgGAM	IgM- and IgA-enriched polyclonal intravenous immunoglobulin
IMV	invasive mechanical ventilation
INR	international normalized ratio
IVIG	intravenous immunoglobulin
LMWH	low-molecular-weight heparin
MAP	mean arterial pressure
OR	odds ratio
PaO_2_/FiO_2_	ratio of arterial partial pressure of oxygen to fraction of inspired oxygen
RCT	randomized controlled trial
RMST	restricted mean survival time
RR	relative risk
RRT	renal replacement therapy
SD	standard deviation
SE	standard error
SOFA	Sequential Organ Failure Assessment
TPE	therapeutic plasma exchange
