# Supplementary material for: IgM- and IgA-Enriched Polyclonal Intravenous Immunoglobulin Therapy in Sepsis: A Single-Center Retrospective Cohort Study
Source: J Clin Med. 2026 Oct 8;15(19):7731. doi: 10.3390/jcm15197731 (PMC13666473; doi:10.3390/jcm15197731)
Supplement: Supplementary file 1 [file jcm-15-07731-s001.zip › jcm-4558497-supplementary.pdf]

Supplementary Materials

IgM- and IgA-Enriched Polyclonal Intravenous Immunoglobulin Therapy in Sepsis: A Single-Center Retrospective Cohort Study

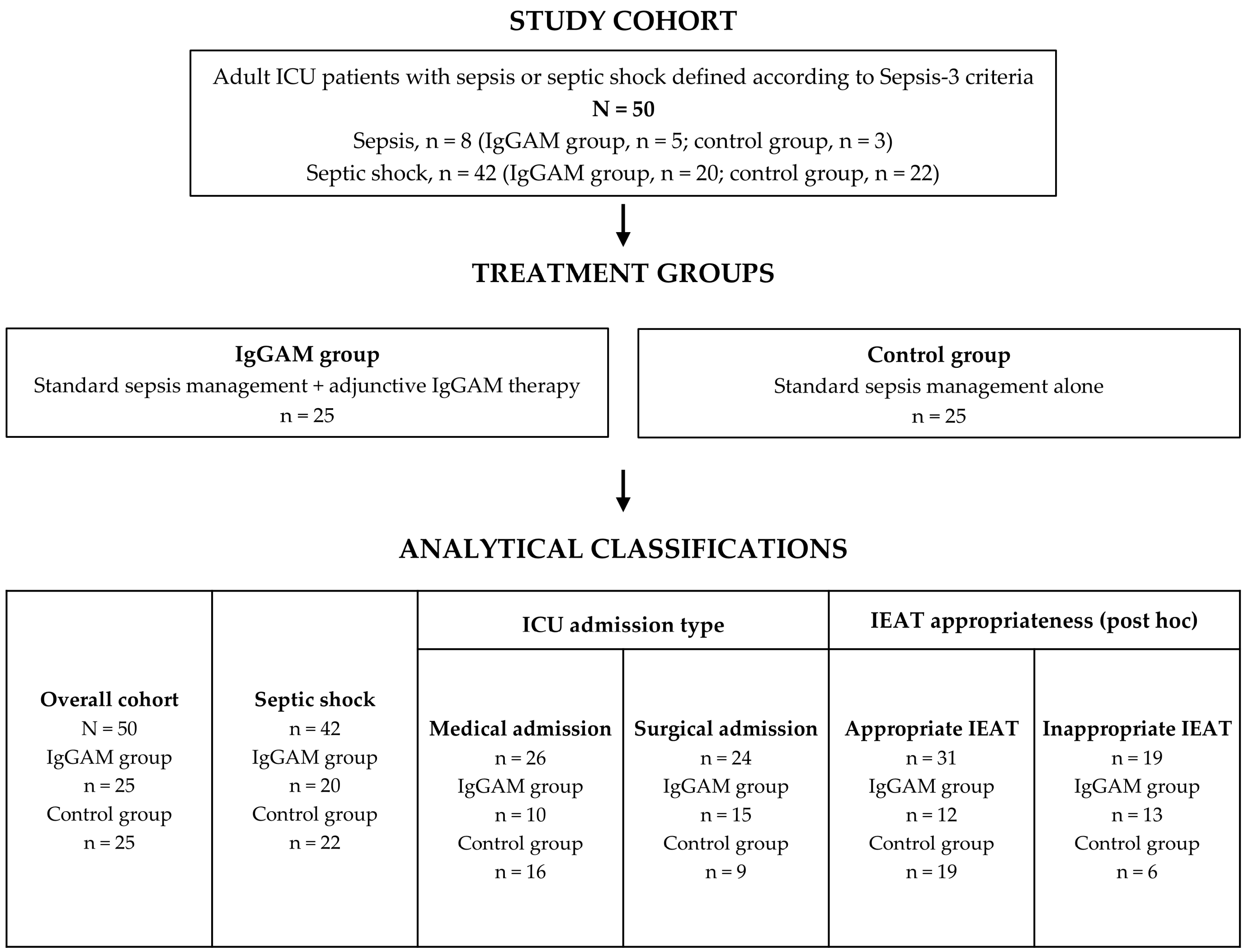

Figure S1. Analytical diagram of the study cohort and subgroup analyses.

The study cohort comprised 50 adult ICU patients with sepsis or septic shock, defined according to Sepsis-3 criteria. Twenty-five patients received standard sepsis management with adjunctive IgGAM therapy, and 25 received standard sepsis management alone. Analyses were performed in the overall cohort, in patients with septic shock, and in subgroups defined by ICU admission type. Subgroup analyses according to IEAT appropriateness were performed post hoc. These analytical classifications do not represent sequential enrollment or exclusion steps.

Abbreviations: ICU, intensive care unit; IEAT, initial empiric antibiotic therapy; IgGAM, IgM- and IgA-enriched polyclonal intravenous immunoglobulin.

Table S1. Distribution of microorganisms isolated during the ICU stay

Isolated microorganism, *n* (%)[a]	IgGAM group (*n* = 25)	Control group (*n* = 25)	*p* value
**Bacteria**	24 (96)	25 (100)	1.000
**Gram-negative bacteria**	24 (96)	24 (96)	1.000
Acinetobacter baumannii	20 (80)	20 (80)	
Pseudomonas aeruginosa	11 (44)	13 (52)	
Klebsiella pneumoniae	13 (52)	11 (44)	
Escherichia coli	8 (32)	10 (40)	
Klebsiella oxytoca	0 (0)	2 (8)	
Citrobacter freundii	0 (0)	2 (8)	
Stenotrophomonas maltophilia	1 (4)	1 (4)	
Legionella pneumophila	1 (4)	0 (0)	
Enterobacter cloacae	0 (0)	1 (4)	
Providencia rettgeri	0 (0)	1 (4)	
Aeromonas sobria	0 (0)	1 (4)	
**Gram-positive bacteria**	21 (84)	20 (80)	1.000
Staphylococcus aureus	10 (40)	6 (24)	
Staphylococcus hominis	9 (36)	11 (44)	
Staphylococcus epidermidis	7 (28)	11 (44)	
Staphylococcus haemolyticus	8 (32)	5 (20)	
Staphylococcus sciuri	1 (4)	2 (8)	
Staphylococcus warneri	1 (4)	0 (0)	
Enterococcus faecium	11 (44)	5 (20)	
Enterococcus faecalis	0 (0)	2 (8)	
Corynebacterium bovis	0 (0)	3 (12)	
Corynebacterium genitalium	0 (0)	1 (4)	
Corynebacterium propinquum	0 (0)	1 (4)	
**Fungi**	20 (80)	16 (64)	0.208
Candida albicans	20 (80)	14 (56)	
Candida glabrata	0 (0)	2 (8)	
Candida parapsilosis	1 (4)	1 (4)	
Trichosporon asahii	2 (8)	2 (8)	

[a] Many patients had polymicrobial culture results; therefore, percentages may exceed 100%. *p* values are reported only for broad microorganism categories; species-level data are presented descriptively.

Abbreviations: ICU, intensive care unit; IgGAM, IgM- and IgA-enriched polyclonal intravenous immunoglobulin.

Table S2. APACHE II scores over time by treatment group in the complete-case longitudinal analysis of the overall cohort

Study day	IgGAM group (*n* = 20) median (min–max)	Control group (*n* = 25) median (min–max)	*p* value[a]
1	15 (6–25)	18 (12–37)	0.040
2	14.5 (5–27)	20 (8–36)	0.061
3	14 (4–30)	19 (8–38)	0.031
5	13 (2–29)	20 (9–40)	0.032
7	12 (6–33)	17 (7–36)	0.056
10	13.5 (3–34)	19 (10–38)	0.076
14	11 (3–40)	20 (6–42)	0.045
**Within-group change over time[b]**	$p = 0.852$	$p = 0.131$	

The longitudinal analysis was restricted to patients with APACHE II measurements available at all seven prespecified study days. Descriptive statistics and between-group comparisons at each study day are based on this complete-case population. Full-cohort study day 1 values are reported in Table 2 of the main manuscript.

[a] Between-group *p* values were calculated using the Mann–Whitney U test at each study day.

[b] Within-group changes over time were assessed using the Friedman test.

Abbreviations: APACHE II, Acute Physiology and Chronic Health Evaluation II; IgGAM, IgM- and IgA-enriched polyclonal intravenous immunoglobulin.

Table S3. SOFA scores over time by treatment group in the complete-case longitudinal analysis of the overall cohort

Study day	IgGAM group (n = 20) median (min–max)	Control group (n = 25) median (min–max)	p value[a]
1	8.5 (2–18)	10 (3–15)	0.094
2	7.5 (3–18)	11 (3–14)	0.176
3	7 (3–18)	10 (3–15)	0.119
5	5.5 (1–18)*	10 (3–15)	0.066
7	7 (2–19)	9 (3–16)	0.176
10	7.5 (1–20)	8 (2–16)	0.325
14	5.5 (1–20)*	10 (2–18)	0.429
**Within-group change over time[b]**	p = 0.017	p = 0.616	

The longitudinal analysis was restricted to patients with SOFA measurements available at all seven prespecified study days. Descriptive statistics and between-group comparisons at each study day are based on this complete-case population. Full-cohort study day 1 values are reported in Table 2 of the main manuscript.

[a] Between-group p values were calculated using the Mann–Whitney U test at each study day.

[b] Within-group changes over time were assessed using the Friedman test. An asterisk (*) indicates $p < 0.01$ versus study day 1 in Bonferroni-adjusted post hoc comparisons within the IgGAM group.

Abbreviations: IgGAM, IgM- and IgA-enriched polyclonal intravenous immunoglobulin; SOFA, Sequential Organ Failure Assessment.

Table S4. SOFA scores over time by treatment group in the complete-case longitudinal analysis of the post hoc subgroup receiving appropriate initial empiric antibiotic therapy

Study day	IgGAM group (*n* = 11) median (min–max)	Control group (*n* = 19) median (min–max)	*p* value[a]
1	8 (3–11)	10 (3–15)	0.029
2	7 (3–12)	11 (3–14)	0.047
3	6 (3–15)	11 (3–15)	0.026
5	4 (1–15)*	11 (3–15)	0.011
7	4 (2–12)*	9 (3–15)	0.062
10	6 (1–12)	8 (2–16)	0.136
14	5 (2–12)	11 (2–16)	0.073
**Within-group change over time[b]**	$p = 0.006$	$p = 0.443$	

The longitudinal analysis was restricted to patients with SOFA measurements available at all seven prespecified study days. Descriptive statistics and between-group comparisons at each study day are based on this complete-case population. Study day 1 values for the full appropriate-IEAT subgroup are reported in Supplementary Table S5. The subgroup analysis according to IEAT appropriateness was performed post hoc.

[a] Between-group *p* values were calculated using the Mann–Whitney U test at each study day.

[b] Within-group changes over time were assessed using the Friedman test. An asterisk (*) indicates $p < 0.01$ versus study day 1 in Bonferroni-adjusted post hoc comparisons within the IgGAM group.

Abbreviations: IEAT, initial empiric antibiotic therapy; IgGAM, IgM- and IgA-enriched polyclonal intravenous immunoglobulin; SOFA, Sequential Organ Failure Assessment.

Table S5. Baseline characteristics by treatment group according to the appropriateness of initial empiric antibiotic therapy

Characteristic	Appropriate IEAT			Inappropriate IEAT		
	IgGAM group (*n* = 12)	Control group (*n* = 19)	*p* value	IgGAM group (*n* = 13)	Control group (*n* = 6)	*p* value
**Demographics and case mix**						
Age, years	43.6 ± 22.5	59.8 ± 20.2	0.062	51.0 ± 20.6	63.8 ± 9.2	0.273
Body weight, kg	78.2 ± 12.7	72.7 ± 11.5	0.261	80.4 ± 19.2	75.8 ± 6.6	0.787
**Sex, n (%)**			0.710			0.177
Female	6 (50.0)	7 (36.8)		7 (53.8)	1 (16.7)	
Male	6 (50.0)	12 (63.2)		6 (46.2)	5 (83.3)	
**ICU admission type, n (%)**			0.262			1.000
Medical	5 (41.7)	13 (68.4)		5 (38.5)	3 (50.0)	
Surgical	7 (58.3)	6 (31.6)		8 (61.5)	3 (50.0)	
**Comorbidities, n (%)**						
Hypertension	4 (33.3)	9 (47.4)	0.484	2 (15.4)	3 (50.0)	0.262
Coronary artery disease	3 (25.0)	7 (36.8)	0.697	2 (15.4)	2 (33.3)	0.557
Congestive heart failure	3 (25.0)	6 (31.6)	1.000	2 (15.4)	2 (33.3)	0.557
Chronic obstructive pulmonary disease	1 (8.3)	0 (0.0)	0.387	3 (23.1)	0 (0.0)	0.517

Characteristic	Appropriate IEAT			Inappropriate IEAT		
	IgGAM group (*n* = 12)	Control group (*n* = 19)	*p* value	IgGAM group (*n* = 13)	Control group (*n* = 6)	*p* value
Diabetes mellitus	3 (25.0)	4 (21.1)	1.000	4 (30.8)	1 (16.7)	1.000
Chronic kidney disease	0 (0.0)	1 (5.3)	1.000	0 (0.0)	2 (33.3)	0.088
Chronic liver disease	2 (16.7)	1 (5.3)	0.543	1 (7.7)	0 (0.0)	1.000
**Study day 1 severity**						
**Sepsis stage, n (%)**			0.350			1.000
Sepsis	3 (25.0)	2 (10.5)		2 (15.4)	1 (16.7)	
Septic shock	9 (75.0)	17 (89.5)		11 (84.6)	5 (83.3)	
APACHE II score	12.5 (5–23)	18 (12–37)	0.004	20 (6–25)	18 (12–31)	0.628
SOFA score	7.5 (3–11)	10 (3–15)	0.015	7 (2–18)	10.5 (3–13)	0.757
**Study day 1 hemodynamic parameters**						
Mean arterial pressure, mmHg	63 (52–97)	63 (46–138)	1.000	64 (50–91)	70.5 (53–125)	0.599
Central venous pressure, mmHg	7 (4–13)	10 (3–14)	0.487	8 (4–18)	7 (5–16)	0.930
**Study day 1 laboratory parameters**						
Platelet count, $10^3/\mu L$	196.5 (20–344)	121 (5–420)	0.441	154 (11–1090)	186 (20–442)	0.726
Creatinine, mg/dL	0.9 (0.4–4.5)	1 (0.2–3.9)	0.700	1.1 (0.4–3)	1 (0.7–2.3)	0.930
Total bilirubin, mg/dL	0.9 (0.3–9.4)	0.9 (0.4–14.4)	0.503	1.1 (0.3–19)	1.1 (0.3–13.3)	0.568
C-reactive protein, mg/L	110 (40–145)	120.5 (24–400)	0.308	80 (13.3–310)	125 (38.7–253)	0.763
Procalcitonin, ng/mL	9 (0.5–58.4)	5.5 (0.3–180)	0.903	9.6 (0.2–63.1)	3.1 (0.8–5.2)	0.073
**Study day 1 arterial blood gas values**						
PaO_2/FiO_2 ratio, mmHg	195.5 (120–337)	223 (66–526)	0.543	182 (54–357)	160 (115–214)	0.539
Lactate, mmol/L	2 (0.4–7)	2.3 (1.3–34)	0.340	2.4 (1.1–19)	2.1 (1.6–4.5)	0.895

Characteristic	Appropriate IEAT			Inappropriate IEAT		
	IgGAM group (n = 12)	Control group (n = 19)	p value	IgGAM group (n = 13)	Control group (n = 6)	p value
**Infection characteristics**						
**Infection sites, n (%)**						
Pulmonary	12 (100.0)	16 (84.2)	0.265	11 (84.6)	5 (83.3)	1.000
Intra-abdominal	6 (50.0)	7 (36.8)	0.710	8 (61.5)	3 (50.0)	1.000
Urinary	3 (25.0)	11 (57.9)	0.138	7 (53.8)	4 (66.7)	1.000
Catheter-related bacteremia	4 (33.3)	12 (63.2)	0.149	7 (53.8)	5 (83.3)	0.333
Primary bacteremia	0 (0.0)	1 (5.3)	1.000	0 (0.0)	1 (16.7)	0.316
**Acquisition type, n (%)**			1.000			1.000
Hospital-acquired	10 (83.3)	16 (84.2)		10 (76.9)	5 (83.3)	
Community-acquired	2 (16.7)	3 (15.8)		3 (23.1)	1 (16.7)	
**Culture status, n (%)**			–			–
Culture-positive	12 (100.0)	19 (100.0)		13 (100.0)	6 (100.0)	
Culture-negative	0 (0.0)	0 (0.0)		0 (0.0)	0 (0.0)	
**Microbiological etiology, n (%)**						
Gram-negative bacteria	11 (91.7)	18 (94.7)	1.000	13 (100.0)	6 (100.0)	1.000
Gram-positive bacteria	9 (75.0)	14 (73.7)	1.000	12 (92.3)	6 (100.0)	1.000
Fungi	10 (83.3)	13 (68.4)	0.433	10 (76.9)	3 (50.0)	0.320

More than one infection site and more than one microbiological category could be identified in the same patient; therefore, percentages within these sections may exceed 100%. Abbreviations: APACHE II, Acute Physiology and Chronic Health Evaluation II; IEAT, initial empiric antibiotic therapy; IgGAM, IgM- and IgA-enriched polyclonal intravenous immunoglobulin; ICU, intensive care unit; PaO_2/FiO_2, ratio of arterial partial pressure of oxygen to fraction of inspired oxygen; SD, standard deviation; SOFA, Sequential Organ Failure Assessment.

Table S6. Follow-up duration and censoring in the Kaplan–Meier survival analyses

Cohort or subgroup	Treatment group	*n*	Observed follow-up* range, days	Deaths, *n*	Censored, n (%)	Censoring times, days
Overall cohort	IgGAM	25	14–260	12	13 (52.0)	15, 18, 30, 47, 57, 58, 60, 66, 67, 72, 100, 180, 260
	Control	25	14–120	14	11 (44.0)	20 (×2), 30, 32, 40 (×2), 47, 50, 62, 84, 120
Septic shock	IgGAM	20	14–222	12	8 (40.0)	18, 30, 57, 58, 60, 72, 100, 180
	Control	22	14–120	13	9 (40.9)	20 (×2), 32, 40, 47, 50, 62, 84, 120
Medical admission	IgGAM	10	15–260	4	6 (60.0)	15, 18, 57, 58, 100, 260
	Control	16	14–84	11	5 (31.3)	20, 40, 47, 50, 84
Surgical admission	IgGAM	15	14–222	8	7 (46.7)	30, 47, 60, 66, 67, 72, 180
	Control	9	15–120	3	6 (66.7)	20, 30, 32, 40, 62, 120
Appropriate IEAT**	IgGAM	12	15–260	3	9 (75.0)	15, 18, 30, 47, 57, 60, 100, 180, 260
	Control	19	14–120	12	7 (36.8)	20, 30, 40 (×2), 47, 62, 120
Inappropriate IEAT**	IgGAM	13	14–72	9	4 (30.8)	58, 66, 67, 72
	Control	6	15–84	2	4 (66.7)	20, 32, 50, 84

* Follow-up was measured from study day 1 to death or the last documented date on which the patient was known to be alive. Patients who remained alive at their last documented follow-up were censored at that time. Censoring times are expressed as days from study day 1; ×2 indicates that two patients were censored at the same time point. ** Subgroup analyses according to IEAT appropriateness were performed post hoc. Abbreviations: IEAT, initial empiric antibiotic therapy; IgGAM, IgM- and IgA-enriched polyclonal intravenous immunoglobulin.
